# Supplementary material for: Pharmacokinetics of Bupivacaine Following Administration by an Ultrasound-Guided Transversus Abdominis Plane Block in Cats Undergoing Ovariohysterectomy
Source: Pharmaceutics. 2022 Jul 25;14(8):1548. doi: 10.3390/pharmaceutics14081548 (PMC9331386; doi:10.3390/pharmaceutics14081548)
Supplement: Supplementary file 1 [file pharmaceutics-14-01548-s001.zip › pharmaceutics-1782613-supplementary.pdf]

pharmaceutics

Supplementary materials: Pharmacokinetics of Bupivacaine following Administration by an Ultrasound-Guided Transversus Abdominis Plane Block in Cats Undergoing Ovariohysterectomy

Marta Garbin, Javier Benito, Hélène L. M. Ruel, Ryota Watanabe, Beatriz P. Monteiro, Petra Cagnardi and Paulo V. Steagall

Table S1. Individual pharmacokinetic parameters for bupivacaine after bilateral *transversus abdominis* plane injection of 2 mg/kg or 2.5 mg/kg (BUPI-2 and BUPI-2.5, respectively) in twelve cats (n = 6/group).

**Parameters**	**Units**	**BUPI-2**					
		**Cat 3**	**Cat 5**	**Cat 6**	**Cat 7**	**Cat 10**	**Cat 12**
C_{max}	ng/mL	600	851	1246	1242	2080	979
T_{max}	min	30	60	30	30	30	20
CL/F	mL/min/kg	8.7	5.4	4.9	4.2	3.6	5.0
$T_{1/2}$	min	264	342	236	233	174	271
$AUC_{0\text{-}last}$	min•µg/mL	158	233	302	350	462	273
$AUMC_{0\text{-}last}$	min•min•µg/mL	31290	46828	56992	67379	79778	55015
$MRT_{0\text{-}last}$	min	197	201	189	192	173	201
C_{480}	ng/mL	203	327	318	383	426	351
**Parameters**	**Units**	**BUPI-2.5**					
		**Cat 1**	**Cat 2**	**Cat 4**	**Cat 8**	**Cat 9**	**Cat 11**
C_{max}	ng/mL	1071	2407	2378	1535	1483	1986
T_{max}	min	60	60	30	60	10	60
CL/F	mL/min/kg	6.5	3.0	4.3	3.8	6.3	5.7
$T_{1/2}$	min	233	280	170	265	211	146
$AUC_{0\text{-}last}$	min•µg/mL	283	589	490	447	306	391
$AUMC_{0\text{-}last}$	min•min•µg/mL	55190	116710	82956	91117	55298	61578
$MRT_{0\text{-}last}$	min	195	198	169	204	180	157
C_{480}	ng/mL	370	836	424	590	399	293

One-compartmental and noncompartmental data analyses.
